# Low *WT1* transcript levels at diagnosis predicted poor outcomes of acute myeloid leukemia patients with t(8;21) who received chemotherapy or allogeneic hematopoietic stem cell transplantation

**DOI:** 10.1186/s40880-016-0110-6

**Published:** 2016-05-19

**Authors:** Ya-Zhen Qin, Yu Wang, Hong-Hu Zhu, Robert Peter Gale, Mei-Jie Zhang, Qian Jiang, Hao Jiang, Lan-Ping Xu, Huan Chen, Xiao-Hui Zhang, Yan-Rong Liu, Yue-Yun Lai, Bin Jiang, Kai-Yan Liu, Xiao-Jun Huang

**Affiliations:** Peking University People’s Hospital, Peking University Institute of Hematology, Beijing Key Laboratory of Hematopoietic Stem Cell Transplantation, No. 11 Xizhimen South Street, Beijing, P. R. China; Haematology Research Center, Division of Experimental Medicine, Department of Medicine, Imperial College London, London, SW7 2AZ UK; Biostatistics Division, Medical College of Wisconsin, Milwaukee, WI 53226 USA; Peking-Tsinghua Center for Life Sciences, Beijing, 100871 P. R. China

**Keywords:** Acute myeloid leukemia, *RUNX1*-*RUNX1T1* transcript level, *WT1* transcript level, *C*-*KIT* mutation, Allogeneic hematopoietic stem cell transplantation

## Abstract

**Background:**

Acute myeloid leukemia (AML) with t(8;21) is a heterogeneous disease. Identifying AML patients with t(8;21) who have a poor prognosis despite achieving remission is important for determining the best subsequent therapy. This study aimed to evaluate the impact of Wilm tumor gene-1 (*WT1*) transcript levels and cellular homolog of the viral oncogene *v*-*KIT* receptor tyrosine kinase (*C*-*KIT*) mutations at diagnosis, and *RUNX1*-*RUNX1T1* transcript levels after the second consolidation chemotherapy cycle on outcomes.

**Methods:**

Eighty-eight AML patients with t(8;21) who received chemotherapy only or allogeneic hematopoietic stem cell transplantation (allo-HSCT) were included. Patients who achieved remission, received two or more cycles of consolidation chemotherapy, and had a positive measureable residual disease (MRD) test result (defined as <3-log reduction in *RUNX1*-*RUNX1T1* transcript levels compared to baseline) after 2–8 cycles of consolidation chemotherapy were recommended to receive allo-HSCT. Patients who had a negative MRD test result were recommended to receive further chemotherapy up to only 8 cycles. *WT1* transcript levels and *C*-*KIT* mutations at diagnosis, and *RUNX1*-*RUNX1T1* transcript levels after the second consolidation chemotherapy cycle were tested.

**Results:**

Patients who had a *C*-*KIT* mutation had significantly lower *WT1* transcript levels than patients who did not have a *C*-*KIT* mutation (6.7% ± 10.6% vs. 19.5% ± 19.9%, *P* < 0.001). Low *WT1* transcript levels (≤5.0%) but not *C*-*KIT* mutation at diagnosis, a positive MRD test result after the second cycle of consolidation chemotherapy, and receiving only chemotherapy were independently associated with high cumulative incidence of relapse in all patients (hazard ratio [HR] = 3.53, 2.30, and 11.49; 95% confidence interval [CI] 1.64–7.62, 1.82–7.56, and 4.43–29.82; *P* = 0.002, 0.034, and <0.001, respectively); these conditions were also independently associated with low leukemia-free survival (HR = 3.71, 2.33, and 5.85; 95% CI 1.82–7.56, 1.17–4.64, and 2.75–12.44; *P* < 0.001, 0.016, and <0.001, respectively) and overall survival (HR = 3.50, 2.32, and 4.34; 95% CI 1.56–7.82, 1.09–4.97, and 1.98–9.53; *P* = 0.002, 0.030, and <0.001, respectively) in all patients.

**Conclusions:**

Testing for *WT1* transcript levels at diagnosis in patients with AML and t(8;21) may predict outcomes in those who achieve remission. A randomized study is warranted to determine whether allo-HSCT can improve prognosis in these patients.

## Background

Patients who have acute myeloid leukemia (AML) with t(8;21) have a better prognosis than those with other cytogenetic subtypes [1, 2]. Nevertheless, 30%–40% of patients with t(8;21) relapse, and many die of advanced leukemia [3–5]. Consequently, identifying AML patients with t(8;21) who have a poor prognosis despite achieving remission is important for determining the best subsequent therapy.

Several variables are associated with outcomes in AML patients with t(8;21) [3, 5–14]. Some variables can be ascertained at diagnosis, some after therapy, and others either at diagnosis or after therapy. Many of these variables are confounded. For example, the association between cellular homolog of the viral oncogene v-KIT receptor tyrosine kinase (*C*-*KIT*) mutation at diagnosis and poorer outcomes after chemotherapy is masked by the predictive value of Runt-related transcription factor 1-RUNX1 translocation partner 1 (*RUNX1*-*RUNX1T1*) transcript levels after achieving remission [12, 13]. This is not surprising since response to therapy is usually a better predictor of outcomes than a measurement at diagnosis.

Wilm tumor gene-1 (*WT1*) is a transcription factor overexpressed in diverse neoplasms, including AML. We and others reported that more than 70% of AML patients with overexpressed *WT1* at diagnosis [15–18]. Studies in mice indicated that, in some settings, *WT1* overexpression was required for the development of leukemia [19]. Whether *WT1* transcript levels at diagnosis predict outcomes of AML patients is controversial [20–26]. No study has examined AML patients with t(8;21) who achieved remission.

In our study, we examined 88 AML patients with t(8;21) who enrolled in the multicenter AML05 trial (registered at http://www.chictr.org as #ChiCTR-OCH-12002406); after achieving remission, these patients received chemotherapy only or allogeneic hematopoietic stem cell transplantation (allo-HSCT) [13]. We evaluated the association between *WT1* transcript levels at diagnosis and therapy outcomes, after adjusting for other potential prognostic variables, including *C*-*KIT* mutations at diagnosis and *RUNX1*-*RUNX1T1* transcript levels after the second consolidation chemotherapy cycle.

## Methods

### Patient selection

Between January 2007 and December 2012, 124 consecutive AML patients with t(8;21) (median age 37 years; age range 14–60 years) from three centers (Peking University People’s Hospital, Beijing No. 6 Hospital, and Beijing Rehabilitation Hospital) were enrolled in the AML05 trial. After one or two cycles of induction chemotherapy, 108 patients achieved complete remission; of these, 101 patients had high-quality RNA samples extracted from bone marrow mononuclear cells at diagnosis.

As we previously reported [13], induction chemotherapy was composed of 1–2 cycles of induction with an anthracycline (either daunorubicin 45 mg/m^2^ or idarubicin 8–10 mg/m^2^ for 3 days) in combination with cytarabine 100 mg/m^2^ for 7 days. The first and second cycles of consolidation chemotherapy included intermediate-dose cytarabine (IDAC 1–2 g/m^2^ every 12 h for 3 days) with or without an anthracycline (daunorubicin 45 mg/m^2^ or mitoxantrone 8 mg/m^2^ for 3 days). Post-consolidation chemotherapy was performed as reported previously [13], which included IDAC for 2 cycles, then followed by daunorubicin/idarubicin in combination with cytarabine, homoharringtonine with cytarabine, mitoxantrone with cytarabine, or aclamycin with cytarabine. Among the 101 patients, 1 received no further therapy, 42 received 1–6 cycles of post-consolidation chemotherapy, and 13 received 1–4 cycles of post-consolidation chemotherapy followed by autologous-hematopoietic stem cell transplantation (auto-HSCT); the remaining 45 received no post-consolidation chemotherapy (*n* = 7) or 1–6 courses of post-consolidation chemotherapy (*n* = 38), followed by allo-HSCT from a human leukocyte antigen (HLA)-identical sibling (*n* = 22), a HLA haplotype-matched relative (*n* = 19), or a HLA-matched unrelated donor (*n* = 4). Therapy recommendation was based on the results of measureable residual disease (MRD) testing [27] after 2–8 cycles of consolidation chemotherapy. The real treatment selection was based on both physician’s recommendation and patient’s preference.

RNA extraction, real-time quantitative polymerase chain reaction (RQ-PCR) testing, and *C*-*KIT* mutation testing were performed at Peking University Institute of Hematology. The cutoff date for follow-up was October 31, 2014. We did not consider *WT1* transcript levels at diagnosis when deciding what post-consolidation therapy was received by patients who achieved remission and received consolidation chemotherapy.

### Ethics, consent, and permissions

Our study was approved by the Ethics Committee of Peking University People’s Hospital. In accordance with the Declaration of Helsinki, all patients offered signed informed consent to participate in the study.

### RNA extraction and complementary DNA (cDNA) synthesis

Trizol Reagent (Invitrogen, Carlsbad, CA, USA) was used to extract total RNA. A High Capacity cDNA Reverse Transcription Kit (Applied Biosystems, Foster City, CA, USA) was used to synthesize cDNA.

### Detection of *RUNX1*-*RUNX1T1* and *WT1* transcripts

As described previously, TaqMan-based RQ-PCR technology was used to detect *RUNX1*-*RUNX1T1* and *WT1* transcript levels [18, 28–30]. Primer and probe sequences for Abelson (*ABL*) and *RUNX1*-*RUNX1T1* from the report of the Europe against cancer program [31, 32] were used. Primer and probe sequences used for *WT1* detection were performed as those published previously [30, 33]. Transcript levels were calculated as percent of *RUNX1*-*RUNX1T1* or *WT1* transcript copies/*ABL* copies. We previously identified an upper limit of 0.5% of *WT1* transcript level detection in normal bone marrow samples [18].

In a prior study, the median baseline level of *RUNX1*-*RUNX1T1* transcripts was 388% (range 138%–848%) [34]. We defined a positive MRD test result as a less than 3-log reduction in *RUNX1*-*RUNX1T1* transcript level compared to baseline (>0.4%) after the second cycle of consolidation chemotherapy. Although a positive or negative MRD test result after the second cycle of consolidation chemotherapy was used in the univariate and multivariate analyses, therapy recommendations were based on results of MRD testing at each time point.

### Detection of *C*-*KIT* mutations in exons 17 and 8

cDNA was used for PCR to detect *C*-*KIT* mutations in exons 17 and 8 [10]. The PCR products were analyzed by bidirectional sequencing on an ABI 3730 sequencer (Applied Biosystems). Sensitivity of mutation detection was 10%–20%.

### Statistical analyses and definitions

Fisher’s exact test was used to compare the differences in variable frequencies between cohorts. Martingale residual plot and receiver operating characteristic (ROC) curves based on overall mortality were used to determine the potentially optimal *WT1* cutoff levels. Survival functions were estimated using the Kaplan–Meier method and compared by the log-rank test within the same treatment group. The starting point for comparing outcomes between the cohorts was the point when complete remission was declared. Left-truncated analyses were used to eliminate the potential bias caused by the relapse or death which made the patient unable to receive an allo-HSCT. At each study time point in this model, the risk set in the non-transplant cohort consisted of all patients who were still in the study, whereas the risk set in the transplant cohort included only patients whose waiting time to undergo allo-HSCT was shorter than the current study period and who were still in the study.

Univariate probabilities of overall survival (OS) and leukemia-free survival (LFS) were calculated using a left-truncated version of the Kaplan–Meier estimator with 95% confidence intervals (CIs). To accommodate competing risks, cumulative incidence of relapse (CIR) and treatment-related mortality (death in complete remission) were calculated using a left-truncated version of the cumulative incidence function (CIF). To adjust for the differences in baseline characteristics, left-truncated versions of the Cox proportional hazards regression models were used to evaluate the relative risk of patients who received chemotherapy only versus those who received allo-HSCT. The proportionality assumption was tested by adding a time-dependent covariate. A backward stepwise model selection approach was used to identify all significant risk factors. Furthermore, we analyzed the association of variables with clinical outcomes in the chemotherapy-only and allo-HSCT cohorts. Results of the multivariate analysis were confirmed by fitting a Cox model with a time-dependent treatment assignment in which all patients were considered non-transplant patients and were switched to the transplant cohort when the current study time point passed each individual’s transplant time. LFS was measured from the date that complete remission was detected. Events for LFS included relapse or death after achieving complete remission. Patients or their relatives were queried at the date of the last follow-up or censored on the date the patients were last known to be alive. *P* values less than or equal to 0.05 were considered statistically significant. SAS version 9.3 (SAS Institute Inc., Cary, NC, USA) and SPSS version 13.0 (IBM Corporation, Armonk, NY, USA) software packages and GraphPad Prism 5 (GraphPad Software, Inc., La Jolla, CA, USA) were used for data analyses. Standard definitions of complete remission and relapse were used as reported previously [35]. Relapses included those occurred at the bone marrow and/or extra-medullary sites.

## Results

### Patients and outcomes

Among the 101 patients studied, median follow-up time for those who survived was 38 months (range 4–93 months). Twenty-six chemotherapy-only patients, 1 auto-HSCT patient, and 6 allo-HSCT patients experienced a relapse. Twenty-two chemotherapy-only patients, 14 auto-HSCT patients, and 35 allo-HSCT patients were alive at last follow-up. Three-year CIR, LFS, and OS rates are shown in Table 1.

**Table 1:** Unadjusted outcomes at 3 years of acute myeloid leukemia (AML) patients with t(8;21) who achieved complete remission after 1–2 induction chemotherapy cycles

Group	CIR rate	LFS rate	OS rate
Chemotherapy only	71% (63%–80%)	26% (15%–43%)	41% (25%–58%)
Auto-HSCT	8% (0%–71%)	85% (51%–96%)	92% (57%–99%)
Allo-HSCT	16% (10%–22%)	72% (56%–83%)	77% (61%–87%)
All cohorts	37% (25%–50%)	56% (45%–65%)	65% (54%–74%)

*CIR* cumulative incidence of relapse, *LFS* leukemia-free survival, *OS* overall survival, *allo-HSCT* allogeneic hematopoietic stem cell transplantation, *auto-HSCT* autologous-hematopoietic stem cell transplantation

Owing to the small number of auto-HSCT patients (*n* = 13), the 88 patients who received allo-HSCT (*n* = 45) or chemotherapy only (*n* = 43, including 1 who received no further chemotherapy after two cycles of consolidation chemotherapy) were the basis of our subsequent analysis. Patient characteristics at diagnosis are shown in Table 2. In any variable tested, no significant differences were observed between the chemotherapy-only cohort and the allo-HSCT cohort.

**Table 2:** Variables at diagnosis of AML patients with t(8;21) who achieved complete remission and received chemotherapy only and allo-HSCT

Variable	All	Chemotherapy only	Allo-HSCT	*P* value
Total	88	43	45	–
Age (years)	36 (14–60)	42 (14–60)	36 (14–54)	0.36
Males^a^	47 (53%)	19 (44%)	28 (62%)	0.13
WBC (×10^9^/L)	8.3 (1.3–112)	8.1 (1.3–112)	8.6 (1.2–83)	0.41
Blast cells percentage in the bone marrow (%)	46% (18%–87%)	46% (23%–87%)	48% (18%–83%)	0.82
Platelet count (×10^9^/L)	29 (4–187)	30 (5–187)	28 (4–106)	0.38
Other cytogenetic abnormality than t(8;21)^a^	54 (64%)	24 (59%)	30 (70%)	0.36
*RUNX1*-*RUNX1T1* transcript level	466% (97%–2545%)	532% (186%–2545%)	422% (97%–933%)	0.31
*C*-*KIT* mutation^a^	30 (34%)	15 (35%)	15 (33%)	1.00

*RUNX1*-*RUNX1T1* Runt-related transcription factor 1-RUNX1 translocation partner 1, *C*-*KIT* cellular homolog of the viral oncogene *v*-*KIT* receptor tyrosine kinase

^a^These values are presented as number of patients followed by percentage in parentheses; other values are presented as median followed by a range in parentheses

### Determining the best* WT1* transcript breakpoint

The Martingale residuals plot indicated that the potential breakpoint for *WT1* level was 5.0%–10.0%. We identified 5.0% (≤5.0% vs. >5.0%) as the most appropriate *WT1* breakpoint using the maximized partial likelihood method. In this study, 5.0% was the largest likelihood among 5.0%, 8.0%, and 10.0%. The same *WT1* level breakpoint was identified through ROC curve analyses (data not shown). Based on these data, 40 patients with *WT1* transcript levels ≤5.0% at diagnosis were characterized as having low *WT1* transcript levels, and 48 patients were characterized as having high *WT1* transcript levels.

### Relationship between *WT1* transcript level and *C*-*KIT* mutation at diagnosis

**Fig. 1:**
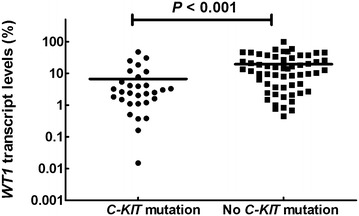
Patients with a cellular homolog of the viral oncogene v-KIT receptor tyrosine kinase (*C*-*KIT*) mutation had significantly lower Wilm tumor gene-1 (*WT1*) transcript levels at diagnosis than patients who did not have a *C*-*KIT* mutation. *Line* represents median *WT1* transcript levels

Among 88 patients, 82 (93.2%) had *WT1* overexpression (transcript level >0.5%) at diagnosis, including 48 (54.5%) had *WT1* overexpression >1-log above the upper limit of normal bone marrow cells (transcript level >5.0%). Thirty patients had *C*-*KIT* mutations detected at diagnosis. Patients who had a *C*-*KIT* mutation had significantly lower *WT1* transcript levels than those who did not have a *C*-*KIT* mutation (mean ± standard deviation: 6.7% ± 10.6% vs. 19.5% ± 19.9%, *P* < 0.001; Fig. 1).

Forty patients had high *WT1* transcript levels and no *C*-*KIT* mutation, 22 patients had low *WT1* transcript levels and a *C*-*KIT* mutation, 18 patients had low *WT1* transcript levels and no *C*-*KIT* mutation, and 8 patients had high *WT1* transcript levels and a *C*-*KIT* mutation. Having low *WT1* transcript levels at diagnosis was significantly associated with *C*-*KIT* mutation (22 of 40 vs. 8 of 48; *P* < 0.001).

### Associations of *WT1* transcript level and *C*-*KIT* mutation at diagnosis with outcomes

**Fig. 2:**
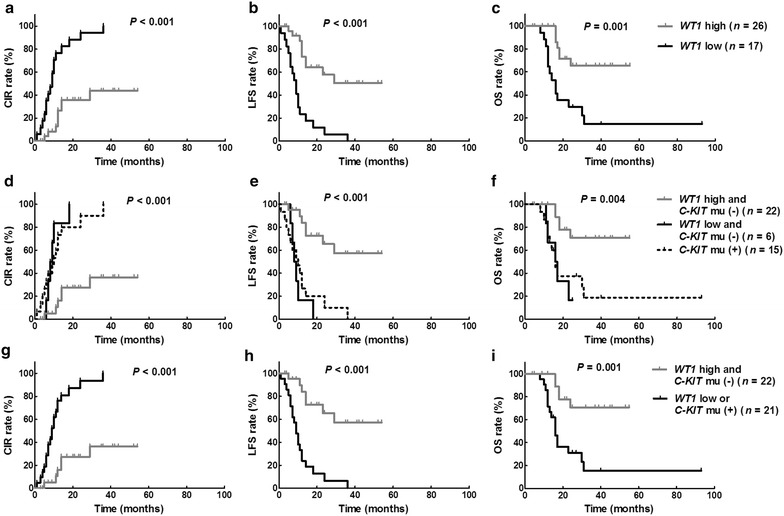
Both *WT1* transcript level and *C*-*KIT* mutation at diagnosis were associated with outcomes in patients who received chemotherapy only. Patients were grouped by *WT1* transcript level and/or *C*-*KIT* mutation at diagnosis. **a**, **d**, **g** cumulative incidence of relapse (CIR) rate (%); **b**, **e**, **h** leukemia-free survival (LFS) rate (%); **c**, **f**, **i** overall survival (OS) rate (%). *Each row* has the same labels that are shown on the *right* of the *row*

Of the 43 patients who received chemotherapy only, 17 who had low *WT1* transcript levels at diagnosis had significantly higher 3-year CIR and lower LFS and OS rates than the 26 patients who had high *WT1* transcript levels at diagnosis (CIR 100% vs. 44% [95% CI 32%–56%], *P* < 0.001; LFS 0% vs. 51% [95% CI 26%–71%], *P* < 0.001; OS 15% [95% CI 3%–36%] vs. 66% [95% CI 41%–82%], *P* = 0.001; Fig. 2). Similarly, the 15 patients who had a *C*-*KIT* mutation at diagnosis had significantly higher 3-year CIR and lower LFS and OS rates than patients without a *C*-*KIT* mutation (CIR 100% vs. 52% [95% CI 30%–70%], *P* = 0.001; LFS 0% vs. 43% [95% CI 22%–63%], *P* = 0.002; OS 19% [95% CI 3%–44%] vs. 57% [95% CI 34%–74%], *P* = 0.018). The 16 patients who had a positive MRD test after the second cycle of consolidation chemotherapy had significantly higher 3-year CIR and lower LFS and OS rates than the 27 patients who had a negative MRD test (CIR 89% [95% CI 79%–95%] vs. 59% [95% CI 39%–75%], *P* < 0.001; LFS 11% [95% CI 1%–35%] vs. 41% [95% CI 19%–61%], *P* < 0.001; OS 25% [95% CI 7%–49%] vs. 58% [95% CI 34%–76%], *P* = 0.001).

Next, we grouped patients undergoing chemotherapy only according to *WT1* transcript level and *C*-*KIT* mutation at diagnosis. The 6 patients who had low *WT1* transcript levels and no *C*-*KIT* mutation had similar 3-year CIR, LFS, and OS rates as the 15 patients who had a *C*-*KIT* mutation (11 with low and 4 with high *WT1* transcript levels at diagnosis; CIR 100% vs. 100%, *P* = 0.491; LFS 0% vs. 0%, *P* = 0.491; OS 16% [95% CI 1%–32%] vs. 19% (95% CI 7%–30%), *P* = 0.640; Fig. 2); these patients were merged in further analyses. The 21 patients who had low *WT1* transcript levels or *C*-*KIT* mutation at diagnosis had a significantly higher 3-year CIR and worse LFS and OS than the 22 patients with high *WT1* transcript levels and no *C*-*KIT* mutation (CIR 100% vs. 36% [95% CI 9%–65%], *P* < 0.001; LFS 0% vs. 57% [95% CI 30%–78%], *P* < 0.001; OS 16% [95% CI 3%–37%] vs. 71% [95% CI 43%–87%], *P* = 0.001; Fig. 2). Based on these data, we classified patients who had low *WT1* transcript levels or a *C*-*KIT* mutation at diagnosis as being *high*-*risk* and those with high *WT1* transcript levels and no *C*-*KIT* mutation at diagnosis as being *low*-*risk*. Using *WT1* transcript levels at diagnosis allowed us to reclassify six patients as *high*-*risk* compared to using *C*-*KIT* mutation state data only to calculate risk.

### Allo-HSCT outcomes

Forty-five patients received allo-HSCT. Patients with low *WT1* transcript levels at diagnosis (*n* = 23) had 3-year CIR, LFS, and OS rates similar to patients with high *WT1* transcript levels at diagnosis (*n* = 22; CIR 13% [95% CI 3%–30%] vs. 15% [95% CI 4%–34%], LFS 64% [95% CI 40%–80%] vs. 80% [95% CI 55%–92%], OS 66% [95% CI 42%–82%] vs. 86% [95% CI 63%–95%]; all *P* > 0.05). Likewise, patients with a *C*-*KIT* mutation at diagnosis (*n* = 15) had 3-year CIR, LFS, and OS rates similar to patients without a *C*-*KIT* mutation at diagnosis (*n* = 30; CIR 14% [95% CI 2%–35%] vs. 14% [95% CI 5%–29%]; LFS 79% [95% CI 49%–93%] vs. 69% [95% CI 48%–82%]; OS 78% [95% CI 47%–92%] vs. 75% [95% CI 55%–87%]; all *P* > 0.05). Patients who had a positive MRD test (*n* = 19) had similar 3-year CIR, LFS, and OS rates as those who had a negative MRD test (*n* = 26; CIR 11% [95% CI 2%–28%] vs. 16% [95% CI 5%–33%]; LFS 68% [95% CI 43%–84%] vs. 76% [95% CI 53%–88%]; OS 74% [95% CI 48%–88%] vs. 79% [95% CI 57%–91%]; all *P* > 0.05).

### Univariate and multivariate analyses in the combined population

*WT1* transcript levels at diagnosis, *C*-*KIT* mutation state at diagnosis, MRD test results after the second cycle of consolidation chemotherapy, and subsequent therapy received (chemotherapy only vs. allo-HSCT) were entered into multivariate analysis for CIR, LFS, and OS. All variables except *C*-*KIT* mutation state at diagnosis were independently associated with these outcomes (Table 3).

**Table 3:** Multivariate analyses of AML patients receiving chemotherapy only and allo-HSCT based on left-truncated Cox model

Variable	No. of patients	HR (95% CI)	*P* value
Relapse			
Therapy			
Allo-HSCT	45	1.00	
Chemotherapy only	43	11.49 (4.43–29.82)	<0.001
*WT1* transcript level^a^			
High	48	1.00	
Low	40	3.53 (1.64–7.62)	0.001
MRD test result^b^			
Negative	53	1.00	
Positive	35	2.30 (1.06–4.97)	0.034
Treatment failure			
Therapy			
Allo-HSCT	45	1.00	
Chemotherapy only	43	5.85 (2.75–12.44)	<0.001
*WT1* transcript level			
High	48	1.00	
Low	40	3.71 (1.82–7.56)	<0.001
MRD test result			
Negative	53	1.00	
Positive	35	2.33 (1.17–4.64)	0.016
Mortality			
Therapy			
Allo-HSCT	45	1.00	
Chemotherapy only	43	4.34 (1.98–9.53)	<0.001
*WT1* transcript level			
High	48	1.00	
Low	40	3.50 (1.56–7.82)	0.002
MRD test result			
Negative	53	1.00	
Positive	35	2.32 (1.09–4.97)	0.030

*HR* hazard ratio, *CI* confidence interval, *WT1* Wilm tumor gene-1, *MRD* measureable residual disease, *RUNX1*-*RUNX1T1* *runt*-*related transcription factor 1*-*RUNX1 translocation partner 1*

^a^Patients with *WT1* transcript levels ≤5.0% and >5.0% at diagnosis were characterized as having low and high *WT1* transcript levels, respectively

^b^A less than and no less than 3-log reduction in *RUNX1*-*RUNX1T1* transcript level compared to baseline (>0.4% and ≤0.4%) after the second cycle of consolidation chemotherapy were defined as positive and negative MRD test results, respectively

### Comparison of outcomes between allo-HSCT and chemotherapy within different risk groups

Of the 40 patients with low *WT1* transcript levels at diagnosis, the 17 who received chemotherapy only had significantly lower LFS and OS rates than the 23 who received allo-HSCT (LFS: HR = 6.70 [95% CI 2.63–17.05], *P* < 0.001; OS: HR = 4.71 [95% CI 1.83–12.07], *P* = 0.001). Of the 48 patients with high *WT1* transcript levels at diagnosis, the 26 who received chemotherapy only had similar LFS and OS rates as the 22 who received allo-HSCT (LFS: HR = 3.44 [95% CI 0.96–12.41], *P* = 0.059; OS: HR = 2.66 [95% CI 0.63–11.16], *P* = 0.183).

## Discussion

Leukemia is one of the leading causes of cancer death [36]. AML is a type of heterogeneous leukemia. In the current study, we found that *WT1* transcript level at diagnosis was significantly associated with CIR, LFS, and OS in patients with AML and t(8; 21) who achieved remission with conventional therapy. Although others have studied the prognostic effect of *WT1* at diagnosis in patients with AML, the results are controversial; some studies reported that high *WT1* transcript levels were associated with a good outcome [20–22], and others reported the contrary [23–26]. These studies included diverse populations and were not restricted to patients who achieved remission.

We also found an association between low *WT1* transcript levels and *C*-*KIT* mutation at diagnosis. Others have reported similar results, but they examined fewer patients [37]. Martingale residual plot and ROC curve analyses indicated that a *WT1* transcript level of ≤5.0 or >5.0% distinguished patients with different CIR, LFS, and OS probabilities.

Multivariate analyses showed that *WT1* transcript levels at diagnosis and results of MRD testing after the second cycle of consolidation chemotherapy, but not *C*-*KIT* mutation at diagnosis, independently associated with CIR, LFS, and OS in patients who received chemotherapy only and in patients who received allo-HSCT. *C*-*KIT* mutation is a widely recognized adverse prognostic factor in AML patients with t(8; 21) [8, 10, 38–42]. Although *WT1* levels at diagnosis associated with *C*-*KIT* mutation, we also noted discordant results. We found that patients with either low *WT1* transcript levels or a *C*-*KIT* mutation at diagnosis had poor prognosis despite achieving remission. However, *WT1* levels at diagnosis were a better predictor of outcome than *C*-*KIT* mutation, and combining them enabled us to reclassify six patients as being at high-risk.

Our previous study suggested that allo-HSCT could improve the prognosis of high-risk patients with t(8;21) after 2–8 cycles of consolidation chemotherapy [13]. Multivariate analyses showed an independent association between therapy type and CIR, LFS, and OS.

Outcomes of patients with low *WT1* transcript levels at diagnosis, whom we defined as *high*-*risk*, were better for those who received allo-HSCT than for those who received chemotherapy only. However, because patients with high *WT1* transcript levels at diagnosis were not randomized to receive either allo-HSCT or chemotherapy only, we cannot be certain that receiving allo-HSCT resulted in better outcomes.

Why low *WT1* transcript levels at diagnosis are associated with poor prognosis in patients with AML is unclear. *WT1* functions are complex, including activation and repression of transcription and oncogenic and tumor-suppressor properties [43–46].

Our study has several important limitations. First, it was not randomized. Second, a substantial number of patients with a positive MRD test result declined their therapy assignment, including some patients who relapsed before allo-HSCT could be done. Third, MRD testing continued to be done at diverse time points following the analysis completed after the second consolidation cycle. To interrogate associations of variables we analyzed with outcomes, we used the results of the test that was conducted after the second consolidation cycle in univariate and multivariate analyses; however, therapy recommendations were sometimes made based on test results from later time points. Fourth, the small sample size of subgroups resulted in relatively low statistical power. These considerations could have biased our results, and a randomized trial is warranted to test the validity of our conclusions.

## Conclusions

Low *WT1* transcript levels at diagnosis are associated with poor outcomes of AML patients with t(8;21) who achieve remission. Patients who received allo-HSCT instead of chemotherapy only had better outcomes. For patients with AML and t(8;21) with low *WT1* transcript levels at diagnosis, a randomized trial of allo-HSCT versus chemotherapy only after consolidation chemotherapy is needed to determine if allo-HSCT improves outcomes.

## Abbreviations

*C*-*KIT* mu (−): without *C*-*KIT* mutation
*C*-*KIT* mu (+): with *C*-*KIT* mutation
